# Incidence, Risk Factors and Outcomes of Urinary Tract Infections among Patients Undergoing Thyroidectomy: Insights from the ACS-NSQIP

**DOI:** 10.1055/s-0044-1788769

**Published:** 2025-01-10

**Authors:** Usama Waqar, Warda Ahmed, Zoha Zahid Fazal, Ahmad Areeb Chaudhry, Haissan Iftikhar, Afsheen Ziauddin, Syed Akbar Abbas

**Affiliations:** 1 Medical College, Aga Khan University, Karachi, Pakistan; 2 Department of Surgery, Emory University School of Medicine and Winship Cancer Institute, Atlanta, GA; 3 Department of Epidemiology and Biostatistics, Rhinology, and Skull Base, University Hospitals Birmingham, Birmingham, United Kingdom; 4 Department of Infection Prevention and Hospital Epidemiology, Aga Khan University Hospital, Karachi, Pakistan; 5 Section of Otolaryngology, Head and Neck Surgery, Department of Surgery, Aga Khan University Hospital, Karachi, Pakistan

**Keywords:** thyroidectomy, urinary tract infection, morbidity, mortality

## Abstract

**Introduction** Urinary tract infections (UTIs) represent a rare postoperative complication following thyroidectomy.

**Objective** This study aimed to assess the clinicodemographic factors associated with the development of UTIs and subsequent outcomes among patients undergoing thyroidectomy.

**Methods** This retrospective study used the National Surgical Quality Improvement Program (NSQIP) database to analyze patients who underwent thyroidectomy from 2005 to 2019. Multivariable logistic regression models were used to identify risk factors and associations of UTIs with postoperative morbidity and mortality.

**Results** In a cohort of 180,373 identified thyroidectomy patients, 0.28% contracted a UTI. Significant risk factors associated with UTIs included age > 60 years (adjusted odds ratio [OR] 2.187, 95% confidence interval [CI] 1.618–2.956), female gender (OR 1.767, 95% CI 1.372–2.278), American Society of Anesthesiologists (ASA) Classification 3 to 5 (OR 1.463, 95% CI 1.185–1.805), partially (OR 4.267, 95% CI 2.510–7.253) or totally dependent functional health status (OR 9.658, 95% CI 4.170–22.370), pulmonary disease (OR1.907, 95% CI 1.295–2.808), chronic steroid therapy (OR 1.649, 95% CI 1.076–2.527), inpatient procedure (OR 1.507, 95% CI 1.251–1.814), and operative time > 150 minutes (OR 1.449, 95% CI 1.027–2.044). Additionally, UTIs were independently associated with postoperative complications, including pulmonary, vascular, or cardiac complication; stroke; acute renal failure; infectious complications; sepsis; septic shock; pneumonia; prolonged length of stay; unplanned reoperation; and mortality.

**Conclusion** While UTIs are rare after thyroidectomy, they carry a significant burden on patient outcomes. Preoperative optimization of comorbidities and reducing operative times may help mitigate the risk of UTIs. Optimized care for postoperative UTI patients is also recommended to prevent complications and improve outcomes.

## Introduction

Thyroidectomy is a common surgical procedure used to manage both benign and malignant thyroid pathologies. As the incidence of thyroid cancer continues to rise at a rate of over 5% annually, the increasing demand for thyroidectomies could pose a significant surgical burden. 1 Thyroidectomy is generally well-tolerated with a minimal morbidity rate. 2 However, complications such as recurrent laryngeal nerve injury, hematoma, and postoperative hypocalcemia may occur. 3

Urinary tract infections (UTIs) represent a well-known postoperative complication across multiple surgical subspecialties. For instance, UTIs account for 40% of all healthcare-associated infections, making it a significant concern for healthcare professionals. 4 In addition to complicating prognosis for patients, UTIs also incur a significant financial burden. In fact, UTIs have cost over 450 million USD and resulted in more than 13,000 deaths annually in the last decades. 4 5 6 Given these serious implications, it is imperative to mitigate their risk among surgery patients and optimize care for patients with UTIs to reduce associated morbidity and mortality.

The incidence of postoperative UTIs among patients undergoing thyroidectomy is rare, with only 0.28% of cases reported in the literature. 7 As a result, there has been a lack of research on the risk factors and outcomes of UTIs in this patient population. To fill this gap, our study aims to evaluate the incidence, underlying risk factors, and postoperative outcomes of 30-day postoperative UTIs in adult patients undergoing thyroidectomy.

## Methods

This retrospective cohort study was conducted in adherence with the Strengthening the Reporting of Observational Studies in Epidemiology (STROBE) reporting guideline, utilizing the American College of Surgeons National Surgical Quality Improvement Program (ACS-NSQIP) database. The ACS-NSQIP partnering hospitals collect standardized, audited clinical data on patient characteristics, preoperative and intraoperative details, and postoperative complications for a predetermined, random sample of their patients. Postoperative outcomes are evaluated by qualified surgical clinical reviewers at each participating center for up to 30 days after the index operation, regardless of patient discharge status. These reviewers assess the patients' medical records, contact the involved clinicians, and reach out to patients as necessary to obtain the required ACS-NSQIP data elements. As this study utilized already deidentified data, it was exempted from review by the Ethics Review Committee at the Aga Khan University in Pakistan (reference ID: 2021–6794–19517).

### Population

Our study population consisted of all adult patients (age ≥ 18 years) who underwent partial, subtotal/total, or completion thyroidectomies for any indication between January 1 ^st^ , 2005, and December 31 ^st^ , 2019. We identified these patients using current procedural terminology (CPT) codes ( Table 1 ). We excluded patients who underwent emergency surgery and those with primary surgical specialty coded other than general surgery or otolaryngology.

**Table 1:** Included current procedural terminology codes

Procedure	CPT codes
Partial thyroidectomy	60210: Partial total lobectomy 60212: Partial total lobectomy with contralateral subtotal lobectomy 60220: Total thyroid lobectomy, unilateral; with or without isthmusectomy
Total/subtotal thyroidectomy	60225: Total thyroid lobectomy, unilateral; with contralateral subtotal lobectomy, including isthmusectomy 60240: Thyroidectomy, total or complete 60252: Thyroidectomy, total or subtotal for malignancy; with limited neck dissection 60254: Thyroidectomy, total or subtotal for malignancy; with radical neck dissection 60270: Thyroidectomy, including substernal thyroid; sternal split or transthoracic approach 60271: Thyroidectomy, including substernal thyroid; cervical approach
Completion thyroidectomy	60260: Thyroidectomy, removal of all remaining thyroid tissue following previous removal of a portion of thyroid

**Abbreviation:** CPT, current procedural terminology.

### Measures

In the present study, both demographic and preoperative comorbidity variables were examined. Age, gender, and race were the demographic variables, while preoperative comorbidities included diabetes mellitus, functional health status, current smoking status, ventilator dependency, chronic obstructive pulmonary disease (COPD), congestive heart failure, hypertension necessitating medication, acute renal failure, dialysis, and steroid/immunosuppressant use for chronic conditions. Additionally, surgical variables such as American Society of Anesthesiologists (ASA) classification, wound classification, surgical indication, type of thyroidectomy, inpatient/outpatient status, and operative time were analyzed. The surgical indications were further classified as benign or malignant using the International Classification of Diseases, Ninth and Tenth Revisions (ICD-9-CM and ICD-10-CM, respectively).

### Outcomes

The primary outcome of interest in this study was the development of UTIs within 30 days after the operation. Index markers for clinically diagnosing UTI included pyrexia (> 38° C), urinary urgency, frequency, dysuria, or suprapubic discomfort in the presence of a urine culture containing more than 100,000 colonies/mL and a maximum of 2 organism species. Alternatively, patients were required to have 2 of the aforementioned symptoms along with a positive dipstick test for leukocyte esterase or nitrates, pyuria greater than 10 white blood cells/mm ^3^ or greater than 3 white blood cells/hpf of unspun urine, organisms visualized on urine gram stain, 2 urine cultures containing the same uropathogen >100 colonies/mL, or one urine culture containing less than 100,000 colonies/mL in a patient who had been prescribed an antibiotic.

Secondary outcomes of interest included all-cause mortality, surgical site infections (SSIs; superficial, deep, or organ/space), sepsis, septic shock, wound disruption, pneumonia, cerebrovascular accident (CVA) or stroke, cardiac arrest requiring cardiopulmonary resuscitation, myocardial infarction, unplanned reintubation, prolonged postoperative ventilator dependence of > 48 hours, progressive renal insufficiency, acute renal failure requiring dialysis, pulmonary embolism, deep venous thrombosis, and unplanned reoperation. Additionally, unplanned reoperation and prolonged length of stay (> 2 days) were also evaluated. Unplanned reoperation was not limited to the index hospital. This study further analyzed composites of these outcomes, namely any complication, infectious and non-infectious complications.

### Statistical Analysis

Patients were first subdivided into UTI and non-UTI groups, and descriptive statistics were reported. Continuous variables were confirmed to have non-parametric distribution using the Kolmogorov-Smirnov test and were reported using median and interquartile ranges (IQRs), and then compared between the two groups using the Mann-Whitney U test. Categorical variables were described using frequencies and percentages and were compared between the groups using the χ2 tests or Fisher exact tests, as appropriate.

To further assess the factors associated with postoperative UTIs in thyroidectomy patients, binary logistic regression models were utilized. Similarly, multivariable models were computed for secondary outcomes, with the development of UTI as the main explanatory variable. Clinically relevant covariates occurring prior to the outcomes and with *p* < 0.25 on univariate analyses were used to adjust these regression models.

All statistical analyses were performed using two-sided tests with α < 0.05 as the threshold for significance. Adjusted odds ratios (ORs) along with 95% confidence intervals (CIs) were reported. Missing data were included in flowcharts and summary tables, which allowed denominators to remain consistent in calculations. The software used for the analyses was the IBM SPSS Statistics for Windows, version 23.0 (IBM Corp., Armonk, NY USA).

## Results

**Fig. 1:**
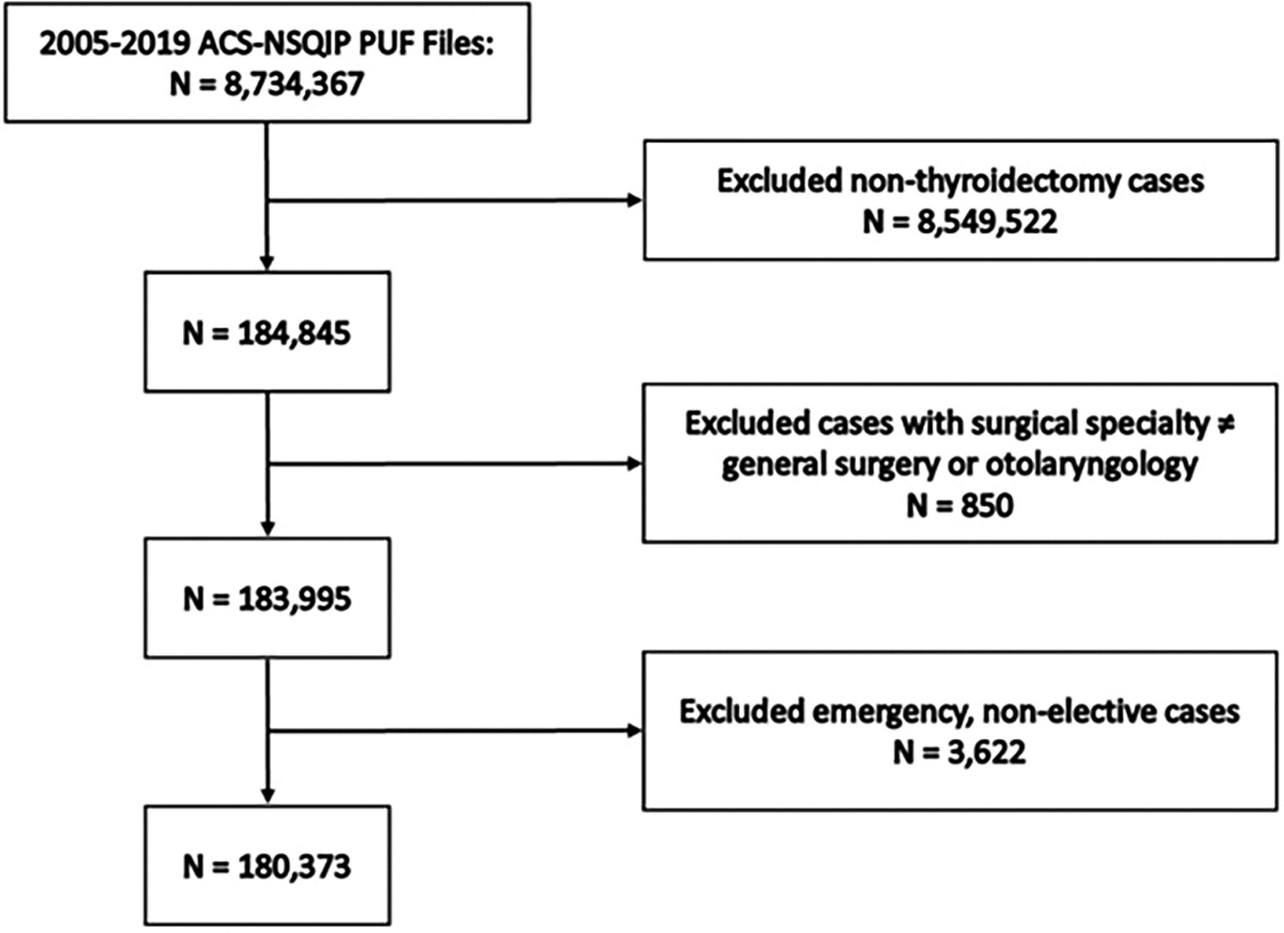
Cohort creation. **Abbreviations:** ACS-NSQIP, American College of Surgeons National Surgical Quality Improvement Program; PUF, participant use data file.

A total of 180,373 thyroidectomy cases were included in the study ( Fig. 1 ), with only 0.28% of patients developing a postoperative UTI. Among these cases, most were female patients, and other sociodemographic characteristics are described in Table 2 . The UTI and non-UTI groups were compared, and the univariate analysis demonstrated several factors to be significantly associated with the incidence of UTIs. These factors included a higher ASA classification, dependent functional health status, diabetes mellitus, chronic steroid therapy, longer operative time, inpatient thyroidectomy, and wound contamination. Moreover, the composite pulmonary and cardiovascular disorders, as well as each of their individual components, were also significantly linked to the occurrence of UTIs.

**Table 2:** Baseline characteristics, comorbidities, and operative variables stratified by urinary tract infection status

Variable	No UTI N = 179,883	UTI N = 490	*p* -value
**Age, in years**			< 0.001
*18–40*	41,878 (23.3%)	69 (14.1%)	
*40–60*	80,993 (45.1%)	155 (31.7%)	
*> 60*	56,858 (31.6%)	265 (54.2%)	
*Missing*	154	1	
Age/years*	52.0 (23.0)	61.0 (23.0)	< 0.001
**Gender**			0.016
*Female*	142,944 (79.5%)	411 (83.9%)	
*Male*	36,939 (20.5%)	79 (16.1%)	
**Race**			0.511
*White*	121,636 (78.5%)	340 (80.6%)	
*Black*	23,362 (15.1%)	54 (12.8%)	
*American Indian or Alaska Native*	699 (0.5%)	3 (0.7%)	
*Asian, Native Hawaiian, or Pacific Islander*	9,182 (5.9%)	25 (5.9%)	
*Missing*	25,004	68	
** BMI (kg/m ^2^ ) **			0.133
*Healthy (18.5-24.9)*	43,195 (24.2%)	108 (22.3%)	
*Underweight (< 18.5)*	1,876 (1.1%)	10 (2.1%)	
*Overweight (25.0–29.9)*	53,829 (30.1%)	151 (31.1%)	
*Obese (30 or higher)*	79,676 (44.6%)	216 (44.5%)	
*Missing*	1,307	5	
BMI (kg/m ^2^ )*	29.1 (9.4)	29.1 (9.1)	0.765
**ASA classification**			< 0.001
*ASA 1–2*	123,710 (68.9%)	251 (51.4%)	
*ASA 3–5*	55,869 (31.1%)	237 (48.6%)	
*Missing*	304	2	
**Functional health status**			< 0.001
*Independent*	178,247 (99.5%)	460 (95.4%)	
*Partially independent*	791 (0.4%)	15 (3.1%)	
*Totally independent*	105 (0.1%)	7 (1.5%)	
*Missing*	740	8	
**Current smoker**	25,604 (14.2%)	60 (12.2%)	0.208
**Diabetes mellitus**	23,139 (12.9%)	97 (19.8%)	< 0.001
**Pulmonary disease**	3,865 (2.1%)	35 (7.1%)	< 0.001
*COPD*	3,823 (2.1%)	30 (6.1%)	< 0.001
*Ventilator dependence*	48 (0.0%)	7 (1.4%)	< 0.001
**Cardiovascular disease**	68,273 (38.0%)	258 (52.7%)	< 0.001
*Hypertension*	68,184 (37.9%)	258 (52.7%)	< 0.001
*Congestive heart failure*	408 (0.2%)	6 (1.2%)	0.001
**Renal disease**	729 (0.4%)	2 (0.4%)	0.727
*Acute renal failure*	72 (0.0%)	1 (0.2%)	0.180
*Currently on dialysis*	695 (0.4%)	1 (0.2%)	1.000
**Chronic steroid therapy**	3,990 (2.2%)	25 (5.1%)	< 0.001
**Wound classification**			0.009
*Clean*	175,857 (97.8%)	468 (95.5%)	
*Clean contaminated*	3,399 (1.9%)	16 (3.3%)	
*Contaminated*	589 (0.3%)	5 (1%)	
*Dirty/infected*	38 (0.0%)	1 (0.2%)	
**Surgical Indication**			0.912
*Benign*	119,986 (66.7%)	328 (66.9%)	
*Malignant*	59,897 (33.3%)	162 (33.1%)	
**Type of thyroidectomy**			0.223
*Partial*	64,756 (36%)	159 (32.4%)	
*Total*	107,650 (59.8%)	307 (62.7%)	
*Completion*	7,477 (4.2%)	24 (4.9%)	
**Inpatient/Outpatient status**			< 0.001
*Outpatient*	113,286 (63.0%)	250 (51.0%)	
*Inpatient*	66,597 (37.0%)	240 (49.0%)	
**Operative time/minutes**			0.001
*Less than 60*	21,405 (11.9%)	54 (11%)	
*60–90*	47,314 (26.3%)	126 (25.7%)	
*90–120*	42,730 (23.8%)	88 (18%)	
*120–150*	28,447 (15.8%)	79 (16.1%)	
*> 150*	39,970 (22.2%)	143 (29.2%)	
*Missing*	17	0	
Operative time/minutes*	103.0 (68.0)	111.0 (85.0)	0.003

**Abbreviations:** ASA, American Society of Anesthesiologists; BMI, body mass index; COPD, chronic obstructive pulmonary disease; UTI, urinary tract infection.

**Note:** * Reported with median and interquartile range; percentages are presented in columns.

After adjusting for clinically relevant covariates, the multivariable logistic regression analysis identified several risk factors associated with the development of postoperative UTI among thyroidectomy patients. These included age > 60 years (adjusted odd ratio [OR]: 2.187, 95% CI: 1.618–2.956), female gender (OR: 1.767, 95% CI: 1.372–2.278), ASA classifications 3 to 5 (OR: 1.463, 95% CI: 1.185–1.805), partially (OR: 4.267, 95% CI: 2.510–7.253) or totally dependent functional health status (OR: 9.658, 95% CI: 4.170–22.370), pulmonary disease (OR: 1.907, 95% CI: 1.295–2.808), chronic steroid therapy (OR 1.649, 95% CI 1.076–2.527), inpatient procedure (OR: 1.507, 95% CI: 1.251–1.814), and operative time greater than 150 minutes (OR: 1.449, 95% CI: 1.027–2.044) ( Table 3 ).

**Table 3:** Multivariable logistic regression analyses for risk factors of 30-day urinary tract infection

Variable	Adjusted OR	*p* -value
**Age/years**		
*18–40*	Reference	–
*40–60*	1,078 (0.803–1,445)	0.618
*> 60*	2,187 (1,618–2,956)	< 0.001
**Gender**		
*Female*	Reference	–
*Male*	0.566 (0.439–0.729)	< 0.001
** BMI (kg/m ^2^ ) **		
*Healthy (18.5–24.9)*	Reference	–
*Underweight (< 18.5)*	1,892 (0.982–3.645)	0.057
*Overweight (25.0–29.9)*	1,048 (0.813–1,351)	0.716
*Obese (30 or higher)*	0.901 (0.704–1,153)	0.408
**ASA classification**		
*ASA 1–2*	Reference	–
*ASA 3–5*	1,463 (1,185–1,805)	< 0.001
**Functional health status**		
*Independent*	Reference	–
*Partially independent*	4,267 (2,510–7,253)	< 0.001
*Totally independent*	9,658 (4,170–22,370)	< 0.001
**Current smoker**	0.851 (0.641–1,129)	0.264
**Diabetes mellitus**	1,128 (0.881–1,444)	0.339
**Pulmonary disease**	1,907 (1,295–2,808)	0.001
**Cardiovascular disease**	1,096 (0.882–1,361)	0.408
**Chronic steroid therapy**	1,649 (1,076–2,527)	0.022
**Wound classification**		
*Clean*	Reference	–
*Clean contaminated*	1,413 (0.840–2,380)	0.193
*Contaminated*	2,545 (0.941–6,886)	0.066
*Dirty/infected*	3,677 (0.387–34,976)	0.257
**Type of thyroidectomy**		
*Partial*	Reference	–
*Total*	0.964 (0.782–1,187)	0.727
*Completion*	1,185 (0.769–1,826)	0.441
**Inpatient/outpatient status**		
*Outpatient*	Reference	–
*Inpatient*	1,507 (1,251–1,814)	< 0.001
**Operative time/minutes**		
*Less than 60*	Reference	–
*60–90*	1,133 (0.816–1,574)	0.455
*90–120*	0.854 (0.599–1,217)	0.382
*120–150*	1,114 (0.770–1,612)	0.565
*> 150*	1,449 (1,027–2,044)	0.035

**Abbreviations:** ASA, American Society of Anesthesiologists; BMI, body mass index; OR, odds ratio.

**Note:** Only cases with complete data on all covariates were included ( *N* = 177,847).

### Complications

The UTI group was found to have significant associations with various postoperative complications compared with the non-UTI group, including any complication, infectious and non-infectious complications, sepsis, septic shock, pneumonia, unplanned reoperation, CVA/stroke with neurological deficit, prolonged length of stay, mortality, and composites of cardiac, vascular, and pulmonary complications ( Table 4 ).

**Table 4:** Postoperative complications at 30-days, stratified by urinary tract infection status

Outcome	No UTI N = 179,883	UTI N = 490	*p* -value
**Any complication**	2,126 (1.2%)	79 (16.1%)	< 0.001
**Non-infectious complication**	1,015 (0.6%)	37 (7.6%)	< 0.001
**CVA/stroke with neurological deficit**	48 (0.0%)	3 (0.6%)	< 0.001
**Cardiac complications**	168 (0.1%)	3 (0.6%)	0.012
*Myocardial infarction*	95 (0.1%)	3 (0.6%)	0.003
*Cardiac arrest requiring CPR*	77 (0.0%)	0 (0.0%)	1.000
**Pulmonary complications**	674 (0.4%)	30 (6.1%)	< 0.001
*Ventilator > 48 hours*	254 (0.1%)	19 (3.9%)	< 0.001
*Unplanned intubation*	579 (0.3%)	23 (4.7%)	< 0.001
**Renal complications**	58 (0.0%)	1 (0.2%)	0.148
*Progressive renal insufficiency*	35 (0.0%)	0 (0.0%)	1.000
*Acute renal failure*	23 (0.0%)	1 (0.2%)	0.063
**Vascular complications**	203 (0.1%)	4 (0.8%)	0.003
*Pulmonary embolism*	109 (0.1%)	2 (0.4%)	0.037
*DVT/thrombophlebitis*	109 (0.1%)	2 (0.4%)	0.037
**Infectious complications**	1,285 (0.7%)	58 (11.8%)	< 0.001
**Surgical site infection**	848 (0.5%)	5 (1.0%)	0.085
*Superficial*	632 (0.4%)	3 (0.6%)	0.249
*Deep*	139 (0.1%)	1 (0.2%)	0.317
*Organ/space*	84 (0.0%)	1 (0.2%)	0.206
**Sepsis**	158 (0.1%)	43 (8.8%)	< 0.001
**Septic shock**	42 (0.0%)	6 (1.2%)	< 0.001
**Wound disruption**	72 (0.0%)	1 (0.2%)	0.180
**Pneumonia**	327 (0.2%)	12 (2.4%)	< 0.001
**Unplanned reoperation**	2,723 (1.5%)	24 (4.9%)	< 0.001
**Prolonged length of stay**			
*No*	170,756 (95%)	406 (83.4%)	< 0.001
*Yes*	9,062 (5%)	81 (16.6%)	
*Missing*	65	3	
**Mortality**	97 (0.1%)	2 (0.4%)	0.030

**Abbreviations:** CPR, cardiopulmonary resuscitation; CVA, cerebrovascular accident; DVT, deep vein thrombosis; UTI, urinary tract infection.

**Note:** Percentages are presented in columns.

After identifying the significant associations between UTIs and various complications, an adjusted analysis was conducted. The results showed that the occurrence of UTIs was strongly associated with any complication (OR: 12.298, 95% CI: 9.471–15.969), acute renal failure (OR: 9.275, 95% CI: 1.223–70.337), and CVA/stroke with neurological deficit (OR: 11.996, 95% CI: 3.652–39.401). In addition, the occurrence of UTIs was also significantly associated with any pulmonary (OR: 10.281, 95% CI: 6.846–15.440), vascular (OR: 3.702, 95% CI: 1.169–11.724), and cardiac complication (OR: 3.476, 95% CI: 1.094–11.045). Infectious complications (OR: 15.561, 95% CI: 11.617–20.844), sepsis (OR: 84.598, 95% CI: 57.738–123.954), septic shock (OR: 32.902, 95% CI: 13.531–80.006), and pneumonia (OR: 8.616, 95% CI: 4.725–15.714) were also significantly associated with postoperative UTIs. Finally, UTIs were found to be associated with prolonged length of stay (OR: 2.914, 95% CI: 2.202–3.855) and unplanned reoperation (OR: 2.818, 95% CI: 1.856–4.279) as well ( Table 5 ).

**Table 5:** Multivariable logistic regression analyses for different 30-day postoperative complications with urinary tract infection as the main explanatory covariate

Outcome	Adjusted OR	*p* -value
**Any complication**	12,298 [9,471–15,969]	< 0.001
**Non-infectious complications**	8,601 [5,944–12,445]	< 0.001
**CVA/stroke with neurological deficit**	11,996 [3,652–39,401]	< 0.001
**Cardiac complication**	3,476 [1,094–11,045]	0.035
*Myocardial Infarction*	6,569 [2,052–21,025]	0.002
*Cardiac arrest requiring CPR*	Could not be computed	**-**
**Pulmonary complication**	10,281 [6,846–15,440]	< 0.001
*Ventilator > 48 hours*	14,841 [8,853–24,879]	< 0.001
*Unplanned intubation*	9,403 [6,014–14,701]	< 0.001
**Renal complication**	3,537 [0.483–25,914]	0.214
*Progressive renal insufficiency*	Could not be computed	**-**
*Acute renal failure*	9,275 [1,223–70,337]	0.031
**Vascular complication**	3,702 [1,169–11,724]	0.026
*Pulmonary embolism*	2,344 [0.324–16,946]	0.399
*DVT/thrombophlebitis*	4,424 [1,076–18,190]	0.039
**Infectious complications**	15,561 [11,617–20,844]	< 0.001
**Surgical site infection**	2,001 [0.824–4,859]	0.125
*Superficial*	1,667 [0.533–5,213]	0.380
*Deep*	2,182 [0.302–15,766]	0.439
*Organ/space*	3,943 [0.542–28,653]	0.175
**Sepsis**	84,598 [57,738–123,954]	< 0.001
**Septic shock**	32,902 [13,531–80,006]	< 0.001
**Wound disruption**	3,850 [0.524–28,265]	0.185
**Pneumonia**	8,616 [4,725–15,714]	< 0.001
**Unplanned reoperation**	2,818 [1,856–4,279]	< 0.001
**Prolonged length of stay**	2,914 [2,202–3,855]	< 0.001
**Mortality**	3,407 [0.820–14,149]	0.092

**Abbreviations:** CVA, cerebrovascular accident; DVT, deep vein thrombosis; OR, odds ratio.

**Notes:** No urinary tract infection was the reference group.

Regression adjusted for age, gender, body mass index, American Society of Anesthesiologists physical status, wound class, operation time, indication, type of thyroidectomy, and inpatient/outpatient surgery.

Only cases with complete data on all covariates and outcomes were included ( *N* = 178,589).

## Discussion

To provide a comprehensive understanding of UTIs among thyroidectomy patients, our study investigated the risk factors and outcomes. Our findings identified several significant risk factors, including age > 60 years, female gender, ASA classification 3 to 5, partially or totally dependent functional health status, pulmonary disease, steroid therapy, inpatient procedure, and an operative time > 150 minutes. Notably, our analysis also revealed that the development of UTIs was associated with an increased likelihood of experiencing various complications, such as pulmonary, vascular, or cardiac complication, stroke, acute renal failure, infectious complications, sepsis, septic shock, pneumonia, prolonged length of stay, unplanned reoperation, and mortality. These results underscore the importance of understanding the risk factors associated with UTIs in this patient population and implementing effective control measures to minimize the occurrence of complications.

Our study found advancing age and female gender to be significant demographic risk factors for UTIs following thyroidectomy. These results are consistent with the well-established association between female gender and UTI risk, as adult women are known to be 30 times more likely to develop UTIs than adult males below 50 years of age. 8 It is worth noting that a previous retrospective analysis reported no difference in UTI rates between young, elderly, and supra-elderly age groups following thyroidectomy. 9 However, the prior study did not perform regression analysis specifically for the development of UTIs and reported only univariate differences, which may explain the disparity in results.

Our analysis revealed several surgical risk factors associated with the development of UTIs following thyroidectomy, including ASA classification 3 to 5, inpatient procedure, and an operative time > 150 minutes. The association between ASA class and postoperative morbidity and mortality has been previously validated. 10 Although a recent study reported an association between dependent status and morbidity following thyroidectomy, it did not specifically analyze the regression model for UTIs, instead using a composite outcome that included UTIs along with other complications. 11 Furthermore, inpatient total thyroidectomies are well-known to be associated with a significantly increased risk of UTIs, which could be due to the higher baseline risk of more complex cases considered for inpatient surgery. 2 Another possible explanation is the faster and more frequent diagnosis of UTIs in the inpatient setting. Similarly, a longer operative time is a known independent risk factor for morbidity following multiple surgical procedures. 12 Taken together, our findings provide insight into the surgical risk factors associated with UTIs following thyroidectomy and can inform targeted interventions to reduce the incidence of this complication.

The importance of UTIs as a target for quality improvement initiatives is highlighted by the Centers for Medicare & Medicaid Services (CMS) and Joint Commission. 13 Our findings have clinical relevance as they demonstrate that UTIs are linked to several postoperative complications and mortality. This is consistent with a previous study that found UTIs to be associated with increased risk of postoperative complications and longer hospital stay in patients undergoing head and neck cancer surgery. 14 The inflammatory process that often accompanies UTIs can lead to urosepsis, a condition that carries a high risk of mortality. Additionally, acute kidney injury resulting from UTIs can lead to septic shock. 15 Notably, UTIs have been associated with up to a 3-fold increase in mortality among patients undergoing colorectal cancer surgery. 16 Despite thyroidectomy being a comparatively simple procedure, the risks of morbidity and mortality following UTIs are concerning.

The current study has several implications. First, our findings highlight the need for a better understanding of modifiable risk factors to prevent the development of UTIs and subsequent morbidity. It is crucial to optimize comorbid diseases associated with UTIs preoperatively. Additionally, further investigation is needed to evaluate and implement surgical techniques and equipment that can shorten operative time. As our results indicate that UTIs increase the risk of postoperative complications, patients who are catheterized or susceptible to UTIs should be counseled to remain vigilant for any signs of developing a UTI. However, the impact of catheterization on the development of UTIs could not be explored in this study, as it is not captured in the ACS-NSQIP. Lastly, following the development of UTIs among thyroidectomy patients, it is imperative to provide optimized care to prevent or adequately manage associated complications and improve patient outcomes.

One major strength of our study is the analysis of a large and diverse sample of thyroidectomy patients from a multi-institutional database, which enhances the generalizability of our findings. However, our study has several limitations that must be acknowledged. Notably, the ACS-NSQIP database lacks information on catheterization and its duration, a known risk factor for UTI development in the inpatient setting. 17 Additionally, we could not differentiate the risk of UTIs based on the method of diagnosis due to the lack of relevant data in the ACS-NSQIP. Our results are limited to the 30-day postoperative period, and we cannot make conclusions beyond this time frame. We also acknowledge the possibility of errors in the database as well as the inherent limitations of retrospective studies, which only allow for the establishment of associations rather than causation. Despite these limitations, our study provides valuable insights into the association between UTIs and postoperative complications following thyroidectomy.

## Conclusion

Although UTIs may be rare among patients undergoing thyroidectomy, they carry a significant morbidity and mortality burden for this cohort. This study has identified the risk factors and outcomes of UTIs among thyroidectomy patients and recommends preoperative optimization of comorbid diseases and reducing operative times as potential measures to mitigate the risk of UTIs. Overall, this study highlights the importance of addressing and managing UTIs in the context of thyroidectomy, which can potentially improve patient outcomes and reduce the burden of postoperative complications.
